# Differences in Cardiovascular Health at the Intersection of Race, Ethnicity, and Sexual Identity

**DOI:** 10.1001/jamanetworkopen.2024.9060

**Published:** 2024-05-01

**Authors:** Nicole Rosendale, Andrew J. Wood, Cindy W. Leung, Anthony S. Kim, Billy A. Caceres

**Affiliations:** 1 Weill Institute for Neurosciences, Department of Neurology, University of California San Francisco; 2 Department of Nutrition, Harvard T.H. Chan School of Public Health, Boston, Massachusetts; 3 Center for Sexual and Gender Minority Health Research, Columbia University School of Nursing, New York, New York

## Abstract

**Importance:**

An understanding of the intersectional effect of sexual identity, race, and ethnicity on disparities in cardiovascular health (CVH) has been limited.

**Objective:**

To evaluate differences in CVH at the intersection of race, ethnicity, and sexual identity using the American Heart Association’s Life’s Essential 8 measure.

**Design, Setting, and Participants:**

This cross-sectional study was conducted from July 27 to September 6, 2023, using National Health and Nutrition Examination Survey data from 2007 to 2016. Participants were noninstitutionalized, nonpregnant adults (aged 18-59 years) without cardiovascular disease or stroke.

**Exposures:**

Self-reported sexual identity, categorized as heterosexual or sexual minority (SM; lesbian, gay, bisexual, or “something else”), and self-reported race and ethnicity, categorized as non-Hispanic Black (hereafter, *Black*), Hispanic, non-Hispanic White (hereafter, *White*), and other (Asian, multiracial, or any other race and ethnicity).

**Main Outcome and Measures:**

The primary outcome was overall CVH score, which is the unweighted mean of 8 CVH metrics, assessed from questionnaire, dietary, and physical examination data. Regression models stratified by sex, race, and ethnicity were developed for the overall CVH score and individual CVH metrics, adjusting for age, survey year, and socioeconomic status (SES) factors.

**Results:**

The sample included 12 180 adults (mean [SD] age, 39.6 [11.7] years; 6147 [50.5%] male, 2464 [20.2%] Black, 3288 [27.0%] Hispanic, 5122 [42.1%] White, and 1306 [10.7%] other race and ethnicity). After adjusting for age, survey year, and SES, Black (β, −3.2; 95% CI, −5.8 to −0.6), Hispanic (β, −5.9; 95% CI, −10.3 to −1.5), and White (β, −3.3; 95% CI, −6.2 to −0.4) SM female adults had lower overall CVH scores compared with their heterosexual counterparts. There were no statistically significant differences for female adults of other race and ethnicity (β, −2.8; 95% CI, −9.3 to 3.7) and for SM male adults of any race and ethnicity compared with their heterosexual counterparts (Black: β, 2.2 [95% CI, −1.2 to 5.7]; Hispanic: β, −0.9 [95% CI, −6.3 to 4.6]; White: β, 1.5 [95% CI, −2.2 to 5.2]; other race and ethnicity: β, −2.2 [95% CI, −8.2 to 3.8]).

**Conclusions and Relevance:**

In this cross-sectional study, CVH differed across race and ethnicity categories in SM females, suggesting that different communities within the larger SM population require tailored interventions to improve CVH. Longitudinal studies are needed to identify the causes of CVH disparities, particularly in Black and Hispanic SM females and inclusive of other racial and ethnic identities.

## Key Points

**Question:**

What differences in cardiovascular health (CVH) exist at the intersection of race, ethnicity, and sexual identity?

**Findings:**

In this cross-sectional study of 12 180 adults using 2007-2016 National Health and Nutrition Examination Survey data, Black, Hispanic, and White sexual minority (SM) female individuals had lower overall CVH scores compared with their heterosexual counterparts. No difference was observed for SM female individuals of other race and ethnicity or SM male individuals across racial and ethnic categories compared with heterosexual individuals.

**Meaning:**

Interventions to improve CVH equity should account for multiple marginalized experiences and address the needs of specific communities, particularly Black and Hispanic SM females.

## Teaser

This cross-sectional study uses National Health and Nutrition Examination Survey data to examine differences in cardiovascular health metrics at the intersection of race, ethnicity, and sexual identity.

## Introduction

Disparities in cardiovascular health (CVH) exist across race and ethnicity categories and in sexual minority (SM; ie, lesbian, gay, and bisexual) adults, but the intersectional effect of multiple marginalized experiences is poorly understood.^1^ Intersectionality is a framework describing how social categories (ie, race, sexual identity) interconnect to create different experiences of discrimination and privilege^2^ and can affect CVH. For example, a 2017 study found that Black SM women had 3 times the prevalence of self-reported stroke compared with Black heterosexual women and 4.5 times the prevalence of self-reported stroke compared with White heterosexual women.^3^ Understanding the effect of multiple marginalized experiences on CVH enables development of tailored interventions to better identify and treat those most at risk.

The American Heart Association’s (AHA’s) measure of ideal CVH, Life’s Essential 8, is composed of 4 health behaviors (diet, physical activity, nicotine exposure, and sleep) and 4 health factors (body weight, blood lipid levels, blood glucose level, and blood pressure)^4^ that have been associated with cardiovascular disease.^5^ A 2023 study using 2007-2016 National Health and Nutrition Examination Survey (NHANES) data found that bisexual female adults had lower CVH scores compared with heterosexual female adults.^6^ The objective of the current study was to investigate the association of sexual identity, race, and ethnicity with CVH. Of note, the term *sexual identity* is used in this article to mirror the language used for this categorization in NHANES; however, we acknowledge that it is not how one identifies that drives inequity but rather how one is categorized in the context of societal power structures.

## Methods

This cross-sectional study, conducted from July 27 to September 6, 2023, was deemed exempt and informed consent was waived by the University of California San Francisco institutional review board because we used publicly available data with no attempt at contacting or identifying deidentified participants. The study was reported according to the Strengthening the Reporting of Observational Studies in Epidemiology (STROBE) reporting guideline.

We conducted a cross-sectional analysis of publicly available NHANES data from 2007 to 2016.^7^ Sexual identity data are not publicly available after 2016, limiting our ability to use more contemporaneous data in our analysis. Full details of the methods were previously published.^6^ We used self-reported questionnaire, physical examination, and dietary recall data to investigate sexual identity differences in CVH scores across racial and ethnic identities among noninstitutionalized, nonpregnant adults (aged 18-59 years) with no history of cardiovascular disease (heart attack, stroke, coronary heart disease, or heart failure) who had complete data for sexual identity, race, ethnicity, and CVH metrics. Participants self-identifying as lesbian, gay, bisexual, or “something else” were categorized as SM due to limited sample size. Self-reported race and ethnicity were categorized as non-Hispanic Black (hereafter, *Black*), Hispanic, non-Hispanic White (hereafter, *White*), and other (Asian, multiracial, or any other race and ethnicity). Following AHA recommendations, respondents received a score of 0 to 100 for each CVH metric, and the overall CVH score was calculated as the unweighted mean of these 8 components, with higher scores corresponding to better CVH.^4^

### Statistical Analysis

NHANES complex survey weights were applied in all analyses. We used an intercategorical approach to the analysis.^8^ This approach was chosen given the hypothesis that those who are marginalized based on race and ethnicity, sexual identity, or both experience barriers to CVH equity compared with White heterosexual individuals. Multiple imputation with chained equations was used for the approximately 5% of respondents with missing demographic data. For univariate analyses, *t* tests and χ^2^ tests were used to compare SM participants across racial and ethnic identities with heterosexual adults. Sex- and race and ethnicity–stratified linear regression models were used to examine differences in CVH metrics and overall CVH score for SM adults across racial and ethnic identities, adjusting for age, survey year, and socioeconomic status (SES) factors (poverty-to-income ratio, educational level, health insurance coverage, and routine place for health care). We also performed sex-stratified linear regression models to examine differences in overall CVH, with White heterosexual adults as the reference category. We used nonstratified linear regression models with an interaction term between race and ethnicity and sexual identity to compare differences in CVH for SM adults of Black, Hispanic, or other race and ethnicity with their heterosexual counterparts. Given that sexual identity data may be missing not at random and therefore multiple imputation of these data may introduce further bias,^9^ we also performed a sensitivity analysis comparing the mean overall CVH score between those with complete sexual identity data and those without sexual identity data across race and ethnicity categories.

Statistical significance was set at 2-sided *P* < .05 for both univariate and multivariable analyses. We did not adjust *P* for multiple comparisons given the descriptive nature of this epidemiologic study, in line with existing recommendations.^10,11^ Analyses were conducted in Stata, version 17 (StataCorp LLC).

## Results

A total of 12 180 participants were included in the analysis (mean [SD] age, 39.6 [11.7] years; 6033 female participants [49.5%]; 6147 male participants [50.5%]). A total of 2464 (20.2%) were Black; 3288 [27.0%], Hispanic; 5122 (42.1%), White; and 1306 (10.7%), other race and ethnicity (eTables 1 and 2 in Supplement 1). Sexual identity data were missing for 6594 participants. Those missing sexual identity data had lower mean (SD) overall CVH scores across race and ethnicity categories compared with those with sexual identity data (Black, 61.4 [13.0] vs 66.3 [13.4]; Hispanic, 63.4 [13.2] vs 69.1 [13.6]; White, 66.7 [13.4] vs 69.9 [14.6]; other race and ethnicity, 71.6 [13.0] vs 74.7 [14.0]).

**Figure 1.**
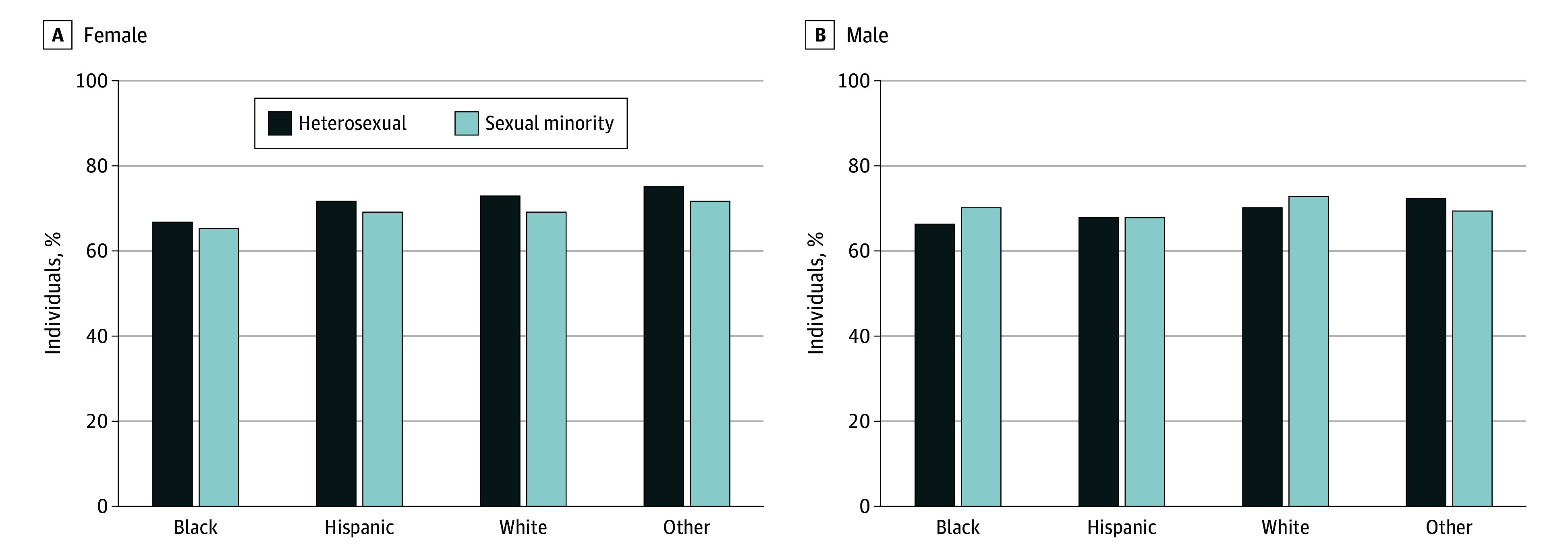
Sexual Identity Differences in Mean Overall Cardiovascular Health Score Across Strata of Race and Ethnicity Among Female and Male Individuals Other race and ethnicity includes those who identified as Asian, multiracial, or any race and ethnicity other than Black, Hispanic, or White. The sexual minority category includes those who identified as lesbian, gay, bisexual, or “something else.” The overall cardiovascular health score is the unweighted mean of the 8 cardiovascular health metrics.

Adjusting for age, survey year, and SES, Black (β, −3.2; 95% CI, −5.8 to −0.6), Hispanic (β, −5.9; 95% CI, −10.3 to −1.5), and White (β, −3.3; 95% CI, −6.2 to −0.4) SM female adults had lower overall CVH scores compared with their heterosexual counterparts (Table 1 and Figure 1; unadjusted analyses are reported in eTable 3 in Supplement 1). There was no significant difference in overall CVH score for female adults of other race and ethnicity (Table 1 and Figure 1) and SM male adults of any race and ethnicity compared with their heterosexual counterparts (Table 2 and Figure 1). Black SM female individuals had lower overall CVH scores (β, −5.7; 95% CI, −8.3 to −3.1) compared with White heterosexual female individuals, but the difference for Hispanic SM female adults was no longer significant (β, −4.3; 95% CI, −8.8 to 0.2). There was no significant difference in overall CVH scores for SM female individuals of a race and ethnicity other than Black or Hispanic compared with White heterosexual female individuals (β, −1.3; 95% CI, −8.2 to 5.6) or in overall CVH scores for SM male individuals of any race and ethnicity compared with White heterosexual male individuals (Black: β, 0.2 [95% CI, −3.5 to 4.0]; Hispanic: β, −1.7 [95% CI, −7.0 to 3.5]; other race and ethnicity: β, −2.7 [95% CI, −8.8 to 3.3]).

**Table 1.** Sexual Identity Differences in CVH Across Strata of Race and Ethnicity Among 6033 Female Individuals

CVH metric	β (95% CI)							
	Adjusted for age and survey year				Adjusted for age, survey year, and SES factors^a^			
	Black	Hispanic	White	Other^b^	Black	Hispanic	White	Other^b^
**Nicotine exposure**								
Heterosexual	1 [Reference]	1 [Reference]	1 [Reference]	1 [Reference]	1 [Reference]	1 [Reference]	1 [Reference]	1 [Reference]
Sexual minority^c^	−20.6 (−32.2 to −9.0)^d^	−10.1 (−22.2 to 1.9)	−20.6 (−29.0 to −12.3)^d^	−16.4 (−34.0 to 1.2)	−16.5 (−28.0 to −5.0)^d^	−11.2 (−23.3 to 0.9)	−14.2 (−22.1 to −6.4)^d^	−7.2 (−26.2 to 11.6)
**Physical activity**								
Heterosexual	1 [Reference]	1 [Reference]	1 [Reference]	1 [Reference]	1 [Reference]	1 [Reference]	1 [Reference]	1 [Reference]
Sexual minority^c^	−1.1 (−10.1 to 8.0)	−1.5 (−13.8 to 10.7)	−0.5 (−7.5 to 6.5)	10.5 (−0.8 to 21.7)	1.1 (−7.7 to 9.9)	−3.2 (−14.9 to 8.5)	1.6 (−5.5 to 8.6)	14.8 (3.7 to 25.8)^d^
**Diet**								
Heterosexual	1 [Reference]	1 [Reference]	1 [Reference]	1 [Reference]	1 [Reference]	1 [Reference]	1 [Reference]	1 [Reference]
Sexual minority^c^	−4.2 (−10.0 to 1.6)	0.8 (−9.3 to 10.9)	−4.0 (−9.8 to 1.8)	−4.3 (−15.6 to 7.1)	−1.3 (−7.0 to 4.5)	0.3 (−9.3 to 9.8)	−0.3 (−5.7 to 5.2)	0.7 (−11.1 to 12.4)
**Sleep**								
Heterosexual	1 [Reference]	1 [Reference]	1 [Reference]	1 [Reference]	1 [Reference]	1 [Reference]	1 [Reference]	1 [Reference]
Sexual minority^c^	−4.8 (−10.6 to 1.1)	−0.1 (−6.0 to 5.8)	−5.6 (−10.3 to −1.0)^d^	−7.1 (−15.0 to 0.9)	−4.1 (−9.8 to 1.7)	−0.5 (−6.4 to 5.5)	−3.0 (−7.9 to 1.8)	−4.3 (−12.9 to 4.3)
**Body mass index**								
Heterosexual	1 [Reference]	1 [Reference]	1 [Reference]	1 [Reference]	1 [Reference]	1 [Reference]	1 [Reference]	1 [Reference]
Sexual minority^c^	−0.6 (−8.2 to 7.0)	−16.3 (−28.9 to −3.6)^d^	−10.4 (−16.6 to −4.2)^d^	−10.3 (−23.8 to 3.3)	0.2 (−7.2 to 7.6)	−17.3 (−29.9 to −4.6)^d^	−8.2 (−14.7 to −1.6)^d^	−8.8 (−20.9 to 3.4)
**Blood pressure**								
Heterosexual	1 [Reference]	1 [Reference]	1 [Reference]	1 [Reference]	1 [Reference]	1 [Reference]	1 [Reference]	1 [Reference]
Sexual minority^c^	−7.0 (−13.7 to −0.4)^d^	−4.7 (−9.5 to 0.02)	−0.2 (−3.5 to 3.1)	−4.5 (−15.2 to 6.3)	−5.1 (−11.5 to 1.3)	−5.1 (−9.8 to −0.4)^d^	0.7 (−2.7 to 4.0)	−3.9 (−14.2 to 6.3)
**Glycemic status^e^**								
Heterosexual	1 [Reference]	1 [Reference]	1 [Reference]	1 [Reference]	1 [Reference]	1 [Reference]	1 [Reference]	1 [Reference]
Sexual minority^c^	−0.3 (−5.8 to 5.2)	−1.4 (−6.4 to 3.5)	−2.8 (−5.7 to 0.2)	−6.0 (−13.0 to 1.1)	0.5 (−4.8 to 5.7)	−2.2 (−7.0 to 2.7)	−1.6 (−4.3 to 1.2)	−4.5 (−11.5 to 2.6)
**Blood lipids**								
Heterosexual	1 [Reference]	1 [Reference]	1 [Reference]	1 [Reference]	1 [Reference]	1 [Reference]	1 [Reference]	1 [Reference]
Sexual minority^c^	−0.5 (−7.5 to 6.6)	−8.4 (−18.3 to 1.5)	−2.6 (−6.7 to 1.4)	−10.0 (−22.2 to 2.1)	−0.4 (−7.5 to 6.7)	−7.9 (−17.2 to 1.4)	−1.1 (−5.1 to 3.0)	−8.9 (−20.5 to 2.6)
**Overall CVH^f^**								
Heterosexual	1 [Reference]	1 [Reference]	1 [Reference]	1 [Reference]	1 [Reference]	1 [Reference]	1 [Reference]	1 [Reference]
Sexual minority^c^	−4.9 (−7.8 to −2.0)^d^	−5.2 (−9.9 to −0.5)^d^	−5.9 (−8.8 to −2.9)^d^	−6.0 (−12.3 to 0.3)	−3.2 (−5.8 to −0.6)^d^	−5.9 (−10.3 to −1.5)^d^	−3.3 (−6.2 to −0.4)^d^	−2.8 (−9.3 to 3.7)

Abbreviations: CVH, cardiovascular health; SES, socioeconomic status.

^a^ SES factors were income-to-poverty ratio, educational level, health insurance coverage, and routine place for health care.

^b^ Includes those who identified as Asian, multiracial, or any race and ethnicity other than Black, Hispanic, or White.

^c^ Includes those who identified as lesbian, gay, bisexual, or “something else.”

^d^ Indicates statistical significance (*P* < .05).

^e^ Assessed using glycosylated hemoglobin.

^f^ Unweighted mean of the 8 CVH metrics.

**Table 2.** Sexual Identity Differences in CVH Across Strata of Race and Ethnicity Among 6147 Male Individuals

CVH metrics	β (95% CI)							
	Adjusted for age and survey year				Adjusted for age, survey year, and SES factors^a^			
	Black	Hispanic	White	Other^b^	Black	Hispanic	White	Other^b^
**Nicotine exposure**								
Heterosexual	1 [Reference]	1 [Reference]	1 [Reference]	1 [Reference]	1 [Reference]	1 [Reference]	1 [Reference]	1 [Reference]
Sexual minority^c^	−9.6 (−22.8 to 3.5)	−16.8 (−33.2 to −0.4)^d^	−6.8 (−18.3 to 4.6)	−17.1 (−48.2 to 13.9)	−13.0 (−25.5 to −0.4)^d^	−20.1 (−35.4 to −4.8)^d^	−9.1 (−19.2 to 1.0)	−12.9 (−43.6 to 17.8)
**Physical activity**								
Heterosexual	1 [Reference]	1 [Reference]	1 [Reference]	1 [Reference]	1 [Reference]	1 [Reference]	1 [Reference]	1 [Reference]
Sexual minority^c^	1.8 (−6.9 to 10.5)	−5.3 (−21.1 to 10.5)	−3.7 (−11.6 to 4.2)	−1.5 (−19.4 to 16.5)	1.7 (−7.1 to 10.4)	−7.2 (−22.6 to 8.3)	−3.7 (−11.7 to 4.2)	−1.2 (−18.6 to 16.2)
**Diet**								
Heterosexual	1 [Reference]	1 [Reference]	1 [Reference]	1 [Reference]	1 [Reference]	1 [Reference]	1 [Reference]	1 [Reference]
Sexual minority^c^	3.1 (−6.8 to 12.9)	11.2 (1.5 to 20.8)^d^	8.8 (1.7 to 15.9)^d^	−17.4 (−36.5 to 1.7)	2.0 (−7.9 to 12.0)	8.8 (0.1 to 17.6)^d^	7.9 (1.2 to 14.7)^d^	−12.0 (−30.1 to 6.1)
**Sleep**								
Heterosexual	1 [Reference]	1 [Reference]	1 [Reference]	1 [Reference]	1 [Reference]	1 [Reference]	1 [Reference]	1 [Reference]
Sexual minority^c^	1.6 (−5.5 to 8.7)	−2.2 (−11.3 to 6.8)	3.9 (−1.2 to 9.0)	−5.6 (−22.8 to 11.5)	1.5 (−5.4 to 8.5)	−1.5 (−10.9 to 7.8)	3.5 (−1.2 to 8.3)	−3.2 (−19.1 to 12.7)
**Body mass index**								
Heterosexual	1 [Reference]	1 [Reference]	1 [Reference]	1 [Reference]	1 [Reference]	1 [Reference]	1 [Reference]	1 [Reference]
Sexual minority^c^	11.7 (2.3 to 21.1)^d^	10.9 (2.2 to 19.6)^d^	6.9 (−1.1 to 14.8)	1.0 (−15.9 to 18.0)	13.1 (3.6 to 22.5)^d^	11.0 (1.9 to 20.1)^d^	6.8 (−1.6 to 15.2)	5.4 (−12.8 to 23.6)
**Blood pressure**								
Heterosexual	1 [Reference]	1 [Reference]	1 [Reference]	1 [Reference]	1 [Reference]	1 [Reference]	1 [Reference]	1 [Reference]
Sexual minority^c^	6.9 (0.5 to 13.2)^d^	0.3 (−5.8 to 6.4)	2.2 (−4.5 to 9.0)	0.03 (−14.9 to 15.0)	6.8 (0.7 to 12.9)^d^	0.3 (−5.8 to 6.4)	1.9 (−4.8 to 8.7)	1.9 (−13.1 to 16.8)
**Glycemic status** ^e^								
Heterosexual	1 [Reference]	1 [Reference]	1 [Reference]	1 [Reference]	1 [Reference]	1 [Reference]	1 [Reference]	1 [Reference]
Sexual minority^c^	5.6 (−1.2 to 12.4)	−2.8 (−12.5 to 6.9)	2.2 (−2.0 to 6.5)	0.7 (−6.8 to 8.3)	6.0 (−1.1 to 13.1)	−3.7 (−13.4 to 6.1)	2.0 (−2.3 to 6.2)	2.6 (−3.4 to 8.5)
**Blood lipids**								
Heterosexual	1 [Reference]	1 [Reference]	1 [Reference]	1 [Reference]	1 [Reference]	1 [Reference]	1 [Reference]	1 [Reference]
Sexual minority^c^	−0.8 (−11.4 to 9.7)	6.9 (−0.9 to 14.6)	3.5 (−2.9 to 9.8)	2.1 (−13.2 to 17.4)	−0.2 (−10.7 to 10.3)	5.5 (−2.3 to 13.4)	2.7 (−3.6 to 9.0)	1.7 (−13.8 to 17.1)
**Overall CVH** ^f^								
Heterosexual	1 [Reference]	1 [Reference]	1 [Reference]	1 [Reference]	1 [Reference]	1 [Reference]	1 [Reference]	1 [Reference]
Sexual minority^c^	2.5 (−1.1 to 6.1)	0.3 (−5.6 to 6.1)	2.1 (−1.7 to 6.0)	−4.7 (−11.2 to 1.8)	2.2 (−1.2 to 5.7)	−0.9 (−6.3 to 4.6)	1.5 (−2.2 to 5.2)	−2.2 (−8.2 to 3.8)

Abbreviations: CVH, cardiovascular health; SES, socioeconomic status.

^a^ SES factors were income-to-poverty ratio, educational level, health insurance coverage, and routine place for health care.

^b^ Includes those who identified as Asian, multiracial, or any race and ethnicity other than Black, Hispanic, or White.

^c^ Includes those who identified as lesbian, gay, bisexual, or “something else.”

^d^ Indicates statistical significance (*P* < .05).

^e^ Assessed using glycosylated hemoglobin.

^f^ Unweighted mean of the 8 CVH metrics.

**Figure 2.**
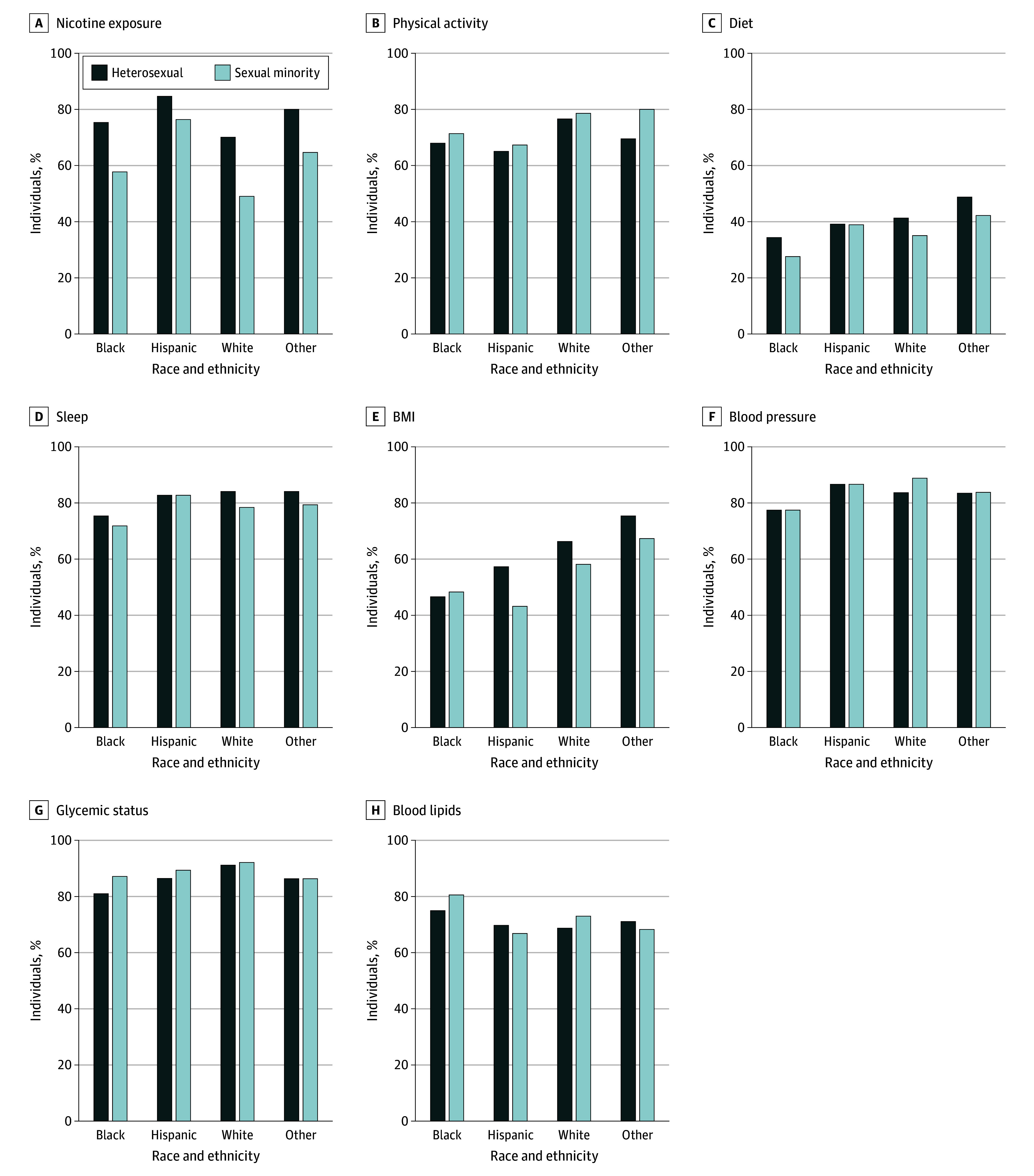
Sexual Identity Differences in Mean Cardiovascular Health Metric Scores Across Strata of Race and Ethnicity Among Female Individuals Other race and ethnicity includes those who identified as Asian, multiracial, or any race and ethnicity other than Black, Hispanic, or White. The sexual minority category includes those who identified as lesbian, gay, bisexual, or “something else.” Glycemic status was assessed using glycosylated hemoglobin. BMI indicates body mass index.

Black SM female individuals had less favorable nicotine scores than their heterosexual counterparts (β, −16.5; 95% CI, −28.0 to −5.0), and Hispanic SM female individuals had less favorable body mass index (BMI) scores (β, −17.3; 95% CI, −29.9 to −4.6) and blood pressure scores (β, −5.1; 95% CI, −9.8 to −0.4) (Table 1 and Figure 2). White SM female individuals had less favorable nicotine exposure scores (β, −14.2; 95% CI, −22.1 to −6.4) and BMI scores (β, −8.2; 95% CI, −14.7 to −1.6) compared with White heterosexual female individuals (Table 1 and Figure 2).

**Figure 3.**
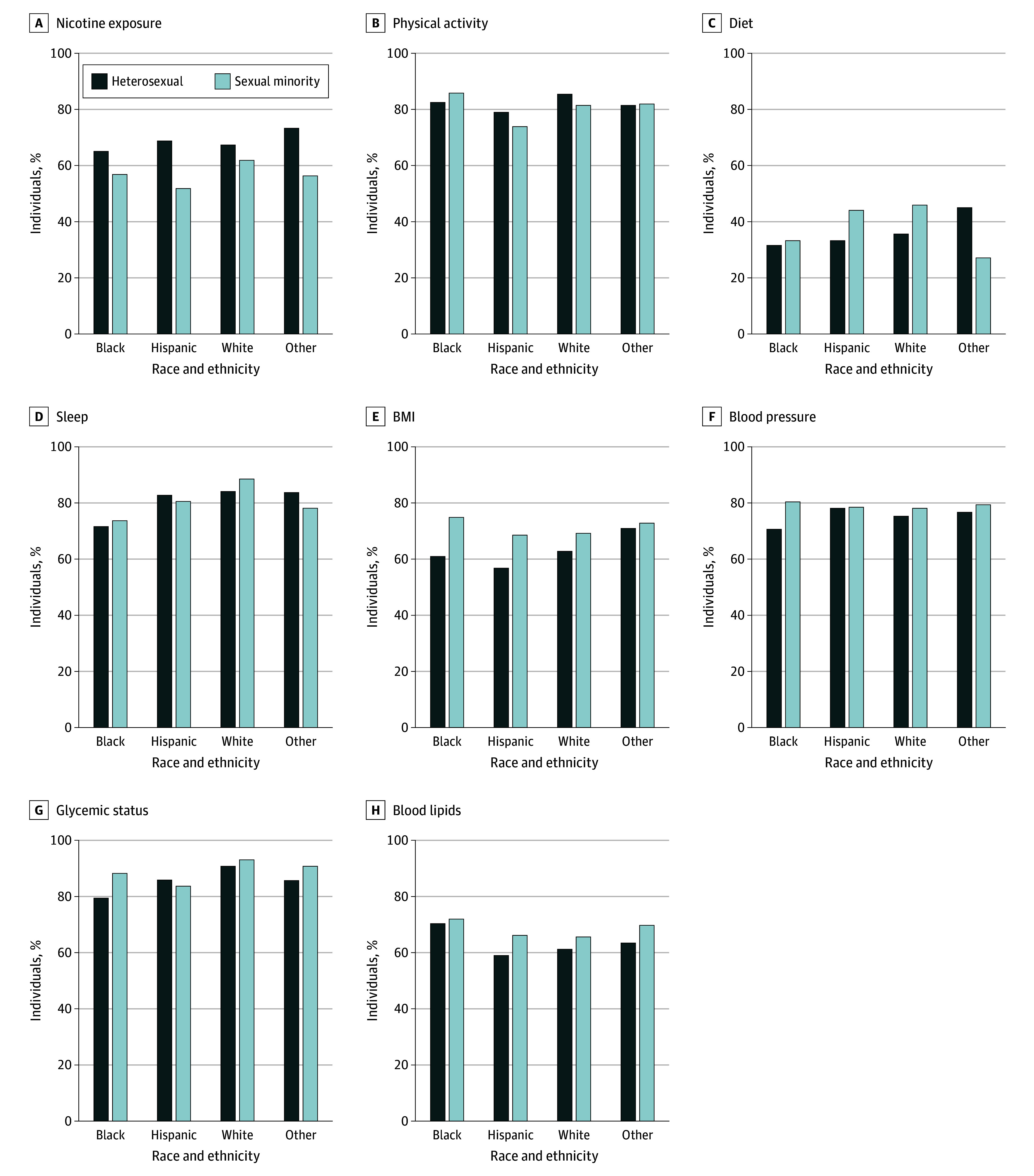
Sexual Identity Differences in Mean Cardiovascular Health Metric Scores Across Strata of Race and Ethnicity Among Male Individuals Other race and ethnicity includes those who identified as Asian, multiracial, or any race and ethnicity other than Black, Hispanic, or White. The sexual minority category includes those who identified as lesbian, gay, bisexual, or “something else.” Glycemic status was assessed using glycosylated hemoglobin. BMI indicates body mass index.

Although there was no statistically significant difference in overall CVH scores for SM male adults compared with heterosexual male adults, Black SM male individuals had less favorable nicotine exposure scores (β, −13.0; 95% CI, −25.5 to −0.4), more favorable BMI scores (β, 13.1; 95% CI, 3.6-22.5), and more favorable blood pressure scores (β, 6.8; 95% CI, 0.7-12.9) compared with Black heterosexual male individuals (Table 2 and Figure 3). Hispanic SM male individuals had less favorable nicotine exposure scores (β, −20.1; 95% CI, −35.4 to −4.8) and more favorable BMI scores (β, 11.0; 95% CI, 1.9-20.1) compared with Hispanic heterosexual male individuals (Table 2 and Figure 3). Both Hispanic and White SM male adults had more favorable diet scores (Hispanic: β, 8.8 [95% CI, 0.1-17.6]; White: β, 7.9 [95% CI, 1.2-14.7]) compared with their heterosexual counterparts (Table 2 and Figure 3). The joint test for significance of the race and ethnicity × sexual identity interaction term across CVH metrics and overall CVH score did not reach statistical significance.

## Discussion

In a nationally representative sample, we found that Black, Hispanic, and White SM female adults had lower overall CVH scores compared with their heterosexual counterparts. Black SM female individuals also had lower overall CVH scores compared with White heterosexual female individuals; there was no association with overall CVH for Hispanic SM female individuals, although this may have been due to limited power from the small sample size. We found no difference in overall CVH in SM male adults across racial and ethnic identities. In the fully adjusted models, overall CVH scores for Black SM female adults were 3.2 points lower compared with Black heterosexual female adults and 5.7 points lower compared with White heterosexual female adults. While the absolute difference between these results is small, the significant disparity when looking across both sexual identity and race and ethnicity categories is consistent with our hypothesis and with the tenets of intersectionality.

Additionally, there were differences in individual CVH metrics; for example, Black and White SM female individuals had less favorable nicotine exposure scores, while Hispanic SM female individuals had less favorable BMI and blood pressure scores compared with heterosexual female individuals of the same race and ethnicity. These differences in CVH metrics suggest the importance of within-group investigations to assess the association of multiple marginalized experiences with health outcomes and is consistent with trends described in the current literature. Sexual minority individuals in marginalized racial and ethnic groups have higher odds of substance use disorder,^12,13^ obesity,^14^ and hypertension compared with White SM people.^15^ The etiology of these disparities is likely multifactorial, including disparate experiences of health care discrimination.^16^

### Limitations

This study has several limitations. The cross-sectional design of NHANES limits causal inference. NHANES does not collect gender identity or expression, limiting our ability to identify transgender, gender nonconforming, and gender diverse people in this analysis. There are also other factors, such as geographic location and direct measures of discrimination, that are not available in NHANES, limiting our ability to explore potential mechanisms for the observed disparities and raising the concern for omitted variable bias. Missingness was high for sexual identity data, and those with missing data had lower CVH scores, meaning that this complete case analysis may overestimate the CVH health of SM people. The sample size was small within sexual identity and race and ethnicity subgroups (ie, Black lesbian female individuals), limiting our ability to perform intracategorical analyses of CVH. Despite these limitations, this study contributes to the current understanding of CVH in SM individuals across racial and ethnic categories.

## Conclusions

This cross-sectional study found differences in CVH across racial and ethnic groups in SM female individuals. This study highlights the importance of incorporating intersectionality into CVH health equity studies and interventions. Longitudinal studies that incorporate mechanistic assessments, such as measures of stress in minority groups, and are inclusive of diverse participants, including those underrepresented in this sample, would be helpful. Tailored interventions to improve the CVH of SM individuals, particularly Black and Hispanic SM female individuals, are needed.

## Notes

Supplement 1. **eTable 1.** Baseline Characteristics Across Strata of Race, Ethnicity, and Sexual Identity Among Female Individuals **eTable 2.** Baseline Characteristics Across Strata of Race, Ethnicity, and Sexual Identity Among Male Individuals **eTable 3.** Unadjusted Regression Results for Female and Male Individuals

Supplement 2.
Data Sharing Statement
