# Supplementary material for: Differences in Cardiovascular Health at the Intersection of Race, Ethnicity, and Sexual Identity
Source: JAMA Netw Open. 2024 May 1;7(5):e249060. doi: 10.1001/jamanetworkopen.2024.9060 (PMC11063800; doi:10.1001/jamanetworkopen.2024.9060)
Supplement: Supplement 1. — eTable 1. Baseline Characteristics Across Strata of Race, Ethnicity, and Sexual Identity Among Female Individuals eTable 2. Baseline Characteristics Across Strata of Race, Ethnicity, and Sexual Identity Among Male Individuals eTable 3. Unadjusted Regression Results for Female and Male Individuals [file jamanetwopen-e249060-s001.pdf]

Supplementary Online Content

Rosendale N, Wood AJ, Leung CW, Kim AS, Caceres BA. Differences in cardiovascular health at the intersection of race, ethnicity, and sexual identity. *JAMA Netw Open*. 2024;7(5):e249060. doi:10.1001/jamanetworkopen.2024.9060

eTable 1. Baseline Characteristics Across Strata of Race, Ethnicity, and Sexual Identity Among Female Individuals
eTable 2. Baseline Characteristics Across Strata of Race, Ethnicity, and Sexual Identity Among Male Individuals
eTable 3. Unadjusted Regression Results for Female and Male Individuals

This supplementary material has been provided by the authors to give readers additional information about their work.

© 2024 Rosendale N et al. *JAMA Network Open*.

eTable 1: Baseline Characteristics Across Strata of Race/Ethnicity and Sexual Identity, among Female Individuals (n= 6,033)

	Heterosexual				Sexual Minority[1]			
	**Black**	**Hispanic**	**White**	**Another Race[2]**	**Black**	**Hispanic**	**White**	**Another Race[2]**
N	1169	1578	2339	543	94	85	178	47
Age, mean (SD)	39.0 (15.4)	37.5 (14.9)	41.5 (9.2)	39.6 (13.8)	31.2 (12.5)*	31.6 (13.4)*	33.9 (9.2)*	32.8 (10.0)*
Family income to poverty ratio, mean (SD)	2.2 (2.1)	2.1 (2.0)	3.3 (1.3)	3.3 (2.0)	1.4 (1.6)*	2.4 (2.0)	2.5 (1.4)*	1.8 (1.7)*
	Weighted %							
Education								
Less than high school	14.6	33.9	8.3	4.1	18.0*	22.5	14.3*	13.4*
High school/GED	23.6	19.3	19.7	9.7	24.4	16.7	22.3	19.8
Some college/AA degree	39.0	31.6	34.1	34.8	48.7	42.1	39.6	36.8
College graduate	22.8	15.3	38.0	51.5	8.9	18.7	23.8	30.1
Health insurance								
Yes	77.3	61.5	86.6	85.5	60.8*	56.4	74.8*	75.8
No	22.7	38.5	13.4	14.5	39.2	43.6	25.2	24.2
Routine place to go for health care								
Yes	90.5	78.0	89.6	84.2	85.2	68.7	80.4*	74.6
No	9.5	22.1	10.4	15.8	14.8	31.3	19.6	25.4

Asterisk (*) denotes statistically significant ($p < 0.05$) difference from heterosexual adult of same race/ethnicity category.

[1] The sexual minority category includes those who identified as lesbian, gay, bisexual, or "something else."

[2] Another race category includes those who identified as Asian, multiracial, and another race/ethnicity other than Hispanic, Black, or White.

© 2024 Rosendale N et al. *JAMA Network Open*.

eTable 2. Baseline Characteristics Across Strata of Race/Ethnicity and Sexual Orientation, among Male Individuals (n= 6,147)

	Heterosexual				Sexual Minority[1]			
	**Black**	**Hispanic**	**White**	**Another Race[2]**	**Black**	**Hispanic**	**White**	**Another Race[2]**
N	1149	1560	2488	691	52	65	117	25
Age, mean (SD)	38.0 (16.5)	36.1 (13.7)	40.3 (9.4)	37.5 (14.1)	33.9 (14.5)*	35.3 (13.3)	40.7 (10.1)	33.0 (11.8)
Family income to poverty ratio, mean (SD)	2.4 (2.2)	2.1 (1.9)	3.4 (1.3)	3.1 (2.1)	2.1 (1.9)	3.1 (2.0)	3.0 (1.4)	2.9 (1.9)
	Weighted %							
Education								
Less than high school	19.3	38.1	8.9	7.3	9.8	18.8*	3.6	6.9
High school/GED	28.5	23.5	23.9	14.2	30.0	16.6	15.8	23.1
Some college/AA degree	36.2	25.7	33.4	26.9	38.8	39.6	38.4	48.4
College graduate	15.9	12.8	33.9	51.6	21.5	25.0	42.2	21.6
Health insurance coverage								
Yes	64.8	50.2	82.9	76.9	67.1	62.4	88.6	68.6
No	35.2	49.8	17.1	23.1	32.9	37.6	11.5	31.4
Routine place to go for health care								
Yes	75.6	59.5	79.7	73.3	84.3	69.6	81.0	59.6
No	24.4	40.5	20.4	26.7	15.7	30.4	19.0	40.4

Asterisk (*) denotes statistically significant ($p < 0.05$) difference from heterosexual adult of same race/ethnicity category.

[1] The sexual minority category includes those who identified as lesbian, gay, bisexual, or “something else.”

[2] Another race category includes those who identified as Asian, multiracial, and another race/ethnicity other than Hispanic, Black, or White.

© 2024 Rosendale N et al. *JAMA Network Open*.

eTable 3: Unadjusted regression results for female and male individuals

	β (95% confidence interval)							
	Female Individuals (n= 6,033)				Male Individuals (n= 6,147)			
CVH Metrics	Black	Hispanic	White	Another Race[1]	Black	Hispanic	White	Another Race[1]
**Nicotine Exposure**								
Heterosexual	[Ref]	[Ref]	[Ref]	[Ref]	[Ref]	[Ref]	[Ref]	[Ref]
Sexual Minority[2]	-17.6* (-29.0, -6.2)	-8.3 (-20.4, 3.8)	-21.0* (-29.5, -12.6)	-15.3 (-32.8, 2.2)	-8.3 (-21.3, 4.8)	-17.2* (-33.5, -0.8)	-5.5 (-17.1, 6.1)	-16.8 (-47.5, 13.9)
**Physical Activity**								
Heterosexual	[Ref]	[Ref]	[Ref]	[Ref]	[Ref]	[Ref]	[Ref]	[Ref]
Sexual Minority	3.4 (-6.0, 12.9)	2.3 (-10.4, 15.0)	2.3 (-4.5, 9.0)	12.4* (0.7, 24.1)	3.3 (-5.0, 11.6)	-5.1 (-20.7, 10.6)	-4.0 (-11.6, 3.6)	0.4 (-17.8, 18.6)
**Diet**								
Heterosexual	[Ref]	[Ref]	[Ref]	[Ref]	[Ref]	[Ref]	[Ref]	[Ref]
Sexual Minority	-6.8* (-12.7, -0.9)	-0.6 (-11.0, 9.8)	-6.5* (-12.1, -0.8)	-6.6 (-18.3, 5.2)	1.7 (-8.0, 11.3)	10.5* (1.1, 20.0)	10.3* (3.1, 17.5)	-17.9 (-36.9, 1.1)
**Sleep**								
Heterosexual	[Ref]	[Ref]	[Ref]	[Ref]	[Ref]	[Ref]	[Ref]	[Ref]
Sexual Minority	-3.6 (-9.3, 2.2)	-0.03 (-5.8, 5.7)	-5.6* (-10.4, -0.9)	-4.8 (-13.6, 3.9)	2.1 (-5.2, 9.4)	-2.2 (-11.3, 6.9)	4.5 (-0.8, 9.7)	-5.6 (-22.6, 11.4)
**BMI**								
Heterosexual	[Ref]	[Ref]	[Ref]	[Ref]	[Ref]	[Ref]	[Ref]	[Ref]
Sexual Minority	1.7 (-6.5, 10.0)	-14.1* (-26.2, -2.0)	-8.1* (-14.8, -1.5)	-7.8 (-21.1, 5.5)	13.9* (3.9, 23.8)	11.1* (2.2, 20.1)	6.4 (-1.3, 14.0)	1.9 (-15.1, 18.9)
**Blood Pressure**								
Heterosexual	[Ref]	[Ref]	[Ref]	[Ref]	[Ref]	[Ref]	[Ref]	[Ref]
Sexual Minority	0.01 (-7.3, 7.3)	0.2 (-4.5, 4.8)	5.2* (1.3, 9.1)	0.3 (-10.4, 11.0)	9.8* (2.0, 17.6)	0.6 (-6.0, 7.1)	2.7 (-3.9, 9.3)	2.8 (-11.4, 17.0)
**Glycemic Status[3]**								
Heterosexual	[Ref]	[Ref]	[Ref]	[Ref]	[Ref]	[Ref]	[Ref]	[Ref]
Sexual Minority	6.2 (-0.2, 12.6)	3.0 (-2.1, 8.1)	1.0 (-2.5, 4.4)	-0.6 (-9.0, 7.9)	8.8* (1.4, 16.3)	-2.2 (-11.3, 6.9)	2.3 (-2.2, 6.7)	5.1 (-6.2, 16.3)
**Blood Lipids**								
Heterosexual	[Ref]	[Ref]	[Ref]	[Ref]	[Ref]	[Ref]	[Ref]	[Ref]
Sexual Minority	5.6 (-1.8, 13.0)	-2.9 (-12.6, 6.9)	4.3 (-0.5, 9.1)	-2.8 (-14.6, 9.0)	1.6 (-9.8, 13.1)	7.1 (-1.2, 15.5)	4.4 (-2.8, 11.7)	6.3 (-12.0, 24.6)

© 2024 Rosendale N et al. *JAMA Network Open*.

**Overall CVH[4]**								
Heterosexual	[Ref]	[Ref]	[Ref]	[Ref]	[Ref]	[Ref]	[Ref]	[Ref]
Sexual Minority	-1.4 (-4.5, 1.8)	-2.6 (-7.4, 2.2)	-3.6* (-6.7, -0.4)	-3.1 (-9.6, 3.3)	4.1 (-0.3, 8.5)	0.3 (-5.6, 6.3)	2.6 (-1.3, 6.6)	-3.0 (-10.3, 4.3)

Asterisk (*) indicates statistical significance, p<0.05

[1] Another race category includes those who identified as Asian, multiracial, and another race/ethnicity other than Hispanic, Black, or White.

[2] The sexual minority category includes those who identified as lesbian, gay, bisexual, or "something else."

[3] Glycemic status was assessed using glycosylated hemoglobin.

[4] Overall CVH is the unweighted average of the 8 CVH metrics.

© 2024 Rosendale N et al. *JAMA Network Open*.
