# Supplementary material for: Differences in Cardiovascular Health at the Intersection of Race, Ethnicity, and Sexual Identity
Source: JAMA Netw Open. 2024 May 1;7(5):e249060. doi: 10.1001/jamanetworkopen.2024.9060 (PMC11063800; doi:10.1001/jamanetworkopen.2024.9060)
Supplement: Supplement 2. — Data Sharing Statement [file jamanetwopen-e249060-s002.pdf]

Data Sharing Statement

Rosendale. Differences in Cardiovascular Health at the Intersection of Race, Ethnicity, and Sexual Identity. *JAMA Netw Open*. Published May 01, 2024.
doi:10.1001/jamanetworkopen.2024.9060

Data
Data available: Yes
Data types: Deidentified participant data
How to access data: https://www.cdc.gov/nchs/nhanes/index.htm
When available: beginning date: 01-01-2013

Supporting Documents
Document types: None

Additional Information
Who can access the data: Data are publicly available
Types of analyses: Any purpose
Mechanisms of data availability: Data are publicly available
